# Adenoid cystic carcinoma of the larynx: Case report and review of literature

**DOI:** 10.1016/j.radcr.2025.04.071

**Published:** 2025-05-12

**Authors:** Fatima Zahra Es-Sahli, Achraf Amine Sbai, Anass Haloui, Drissia Benfadil, Amal Bennani, Azzedine Lachkar, Adil ABDNBI Tsen, Fahd El Ayoubi

**Affiliations:** a Department of Ear Nose and Throat, Mohammed VI University Hospital, Oujda, Morocco; b Laboratory of Epidemiology, Clinical Research and Public Health, Faculty of Medicine and Pharmacy of Oujda, Mohammed the First University, Oujda, Morocco; c Department of Maxillofacial Surgery, Mohammed VI University Hospital, Oujda, Morocco; d Laboratory of Pathological Anatomy, Faculty of Medicine and Pharmacy of Oujda, Mohammed First University, Oujda, Morocco

**Keywords:** Adenoid cystic carcinoma, Perineuronal invasion, Subglottic area, Dyspnea, Laryngectomy, Neck dissection

## Abstract

Adenoid cystic carcinoma (ACC) is a rare malignant neoplasm arising in both major and minor salivary glands. It represents approximately 1% of all head and neck cancers and about 10% of salivary gland tumors. Laryngeal adenoid cystic carcinoma (LACC) is a rare entity that most frequently arises in the subglottic region. It is characterized by slow progression, perineuronal invasion, frequent local recurrences and delayed distant metastasis. Surgical resection remains the gold standard for the treatment of ACC; however, the role of adjuvant radiotherapy and prophylactic neck dissection remains uncertain and is widely debated in the literature.

This case report describes a 60-year-old male patient who presented with progressive dysphagia and worsening dyspnea. Clinical evaluation, including computed tomography (CT), revealed a lesion involving the left glottic and supraglottic regions. Biopsy obtained via direct laryngoscopy confirmed the diagnosis of laryngeal adenoid cystic carcinoma (LACC). The patient subsequently underwent total laryngectomy with adjuvant radiotherapy.

## Introduction

Adenoid cystic carcinomas are malignant tumors that occur in both major and minor salivary glands. Laryngeal ACC, most commonly subglottic, is relatively rare accounting for approximately 0.07%-0.25% of all laryngeal tumors [1]. This neoplasm is notable for its slow progression, perineuronal invasion, and high rate of local recurrences [3,6]. While lymph nodes metastasis is uncommon, distant metastasis can be detected at an early stage [3]. These characteristics underscore the importance of long-term follow-up in affected patients.

Treatment approaches are typically multidisciplinary and may include surgery, radiotherapy, chemotherapy or various combinations [4]. Combined treatment strategies, such as surgery with radiotherapy, are often preferred [4]. Therapeutic neck dissection is indicated for patients with clinical or radiological evidence of positive lymph nodes [16]. However, the management of the clinical negative (cN0) neck remains controversial and prophylactic neck dissection is not routinely performed [16].

In this article, we present a case of this rare laryngeal malignancy in a patient treated with total laryngectomy followed by adjuvant radiotherapy. Published literature regarding ACC of the larynx was also reviewed.

## Case report

A 60-year-old male patient was admitted to our hospital with a 6-month history of progressive dyspnea and a 1-year history of dysphonia with dysphagia. He was a former smoker with no significant past medical history.

**Fig. 1:**
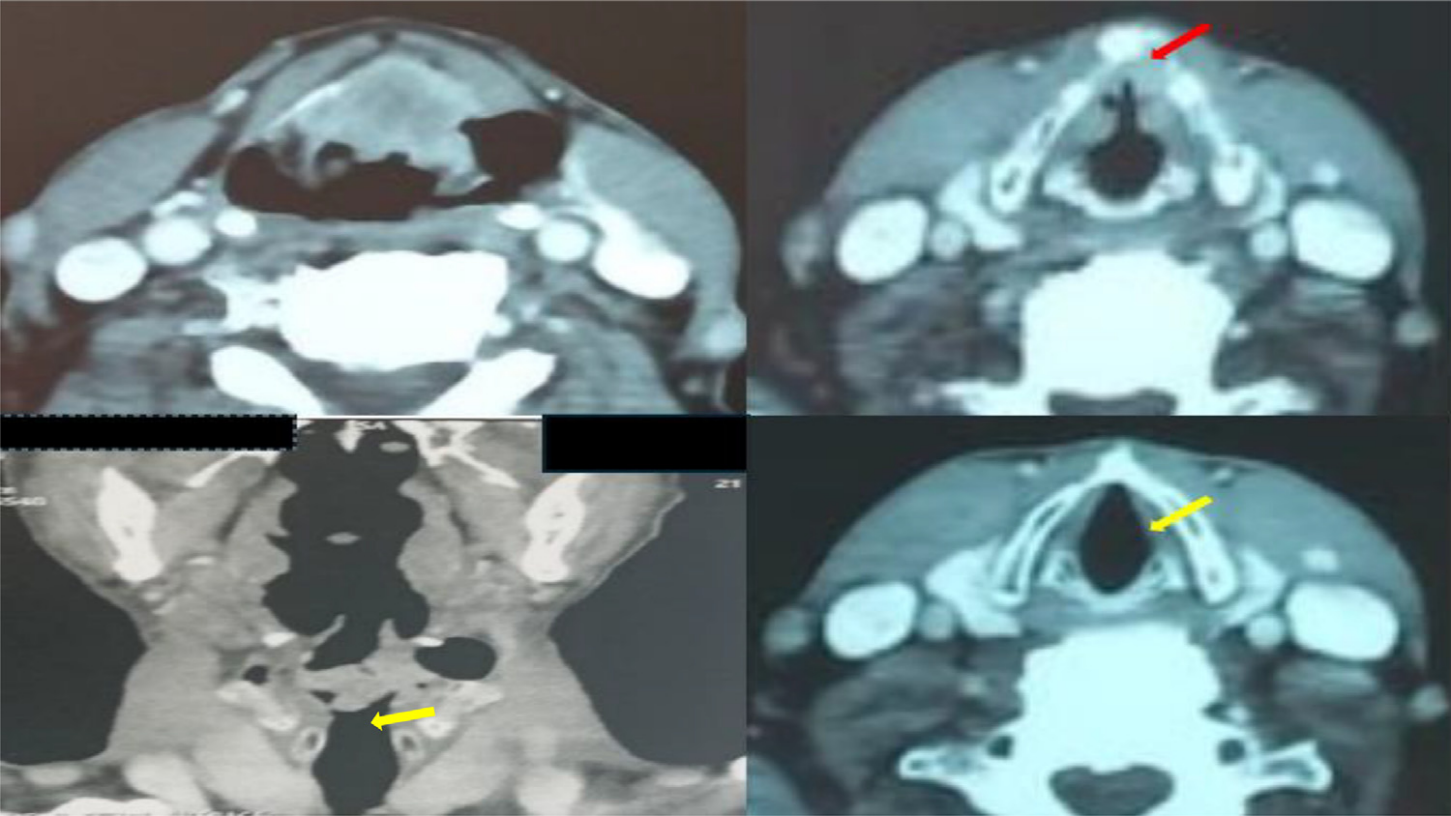
Imaging CT in axial and coronal coupe, showing an irregular process in the glottic and supra-glottic level, reaching the anterior commissure (red arrow). There is no extension to the subglottic area (yellow arrow) or cervical lymph nodes.

Clinical examination including indirect laryngoscopy revealed a bulging supraglottic mass extending to the left vocal cord, which was immobile, resulting in approximately 80% laryngeal stenosis. The subglottic region appeared unremarkable and no palpable cervical lymphadenopathy was noted. Computed tomography of the larynx (Fig. 1) demonstrated an irregular mass in the left supraglottic area, extending inferiorly through the thyroid cartilage to the glottic level, reaching the anterior commissure. There was no evidence of posterior extension to the cricoid cartilage or involvement of the subglottic region. No cervical lymphadenopathy or distant metastases were identified.

**Fig. 2:**
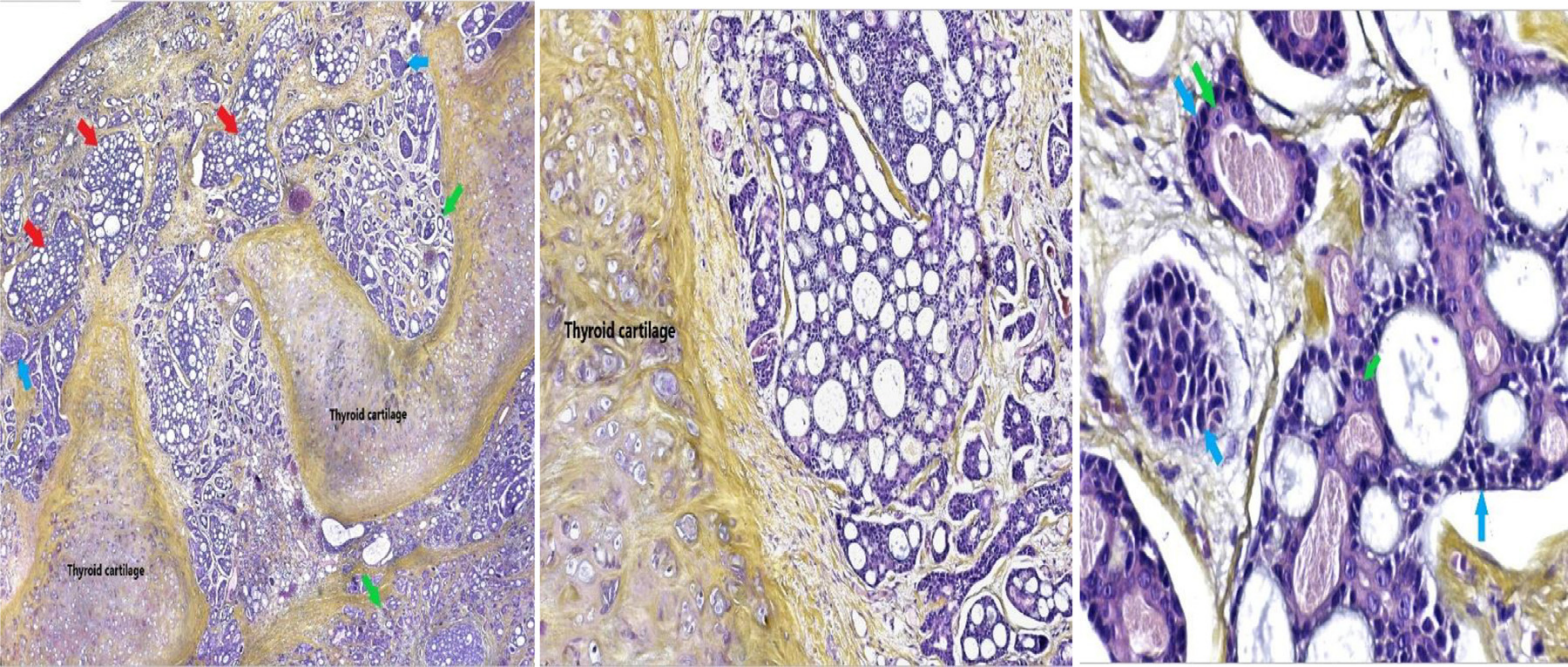
(A) Adenoid cystic carcinoma with predominant cribriform patterns (Red arrow), along with a tubular (Green arrow) and, at a lesser extent, a solid pattern (Blue Arrow). The tumor largely infiltrates the underlying thyroid cartilage. (Hematoxylin-Eosin-Saffron coloration, Magnification x4) (B) Medium power view showing the invasion of thyroid cartilage by the tumor (Hematoxylin-Eosin-Saffron coloration, Magnification x10) (C) The cribriform areas are mainly made of modified myoepithelial cells with indistinct cell borders, amphophilic cytoplasm and uniform round (Blue arrow), The tubular areas are double layered, with luminal ductal cells, (Green arrow) (Hematoxylin-Eosin-Saffron coloration, Magnification x40).

The patient initially underwent a tracheotomy due to severe dyspnea. Biopsy obtained by direct laryngoscopy revealed a cystic adenoid carcinoma of the larynx LACC, with prominent cribriform architecture and evidence perineural invasion (PNI). There were no locoregional or distant metastases and the radioclinical staging was T3N0M0. Therefore, total laryngectomy was performed without neck dissection. Histopathological examination (Fig. 2) confirmed an adenoid cystic carcinoma with a predominant cribriform pattern. Tubular patterns and to lesser extent solid patterns were observed. Extensive perineural invasion was observed, and the tumor had deeply infiltrated the underlying cartilage. Surgical margins were negative, and the final pathological staging was pT3N0M0.

Given the large tumor size and extensive perineural invasion, the patient was scheduled to receive adjuvant radiotherapy. Unfortunately, he was lost to follow-up after completing only a few sessions.

## Discussion

Minor salivary gland carcinomas account for less than 1% of all malignant laryngeal neoplasms [5]. Among these, adenoid cystic carcinoma (ACC) is the most common histologic subtype. Its distribution across laryngeal subsites correlates with the density of submucosal salivary glands, which are most abundant in the subglottic region [1]. Consequently, up to 64% of laryngeal ACCs arise in the subglottic region, followed by the supraglottic (25%), transglottic (6%), and glottic (5%) regions [3]. There are no clear risk factors that predispose patients to this malignancy and smoking does not affect appear to influence the incidence of this malignancy [5,6].

Histopathologically, adenoid cystic carcinoma manifests in 3 distinct patterns: tubular, cribriform, and solid. The solid pattern is the most frequently observed and is associated with a poorer prognosis [7,8]. Notably, these histological patterns may coexist within the same tumor [8], as observed in the present case. This tumor is characterised by perineural invasion PNI, high invasiveness and haematogenous metastasis [19]. Perineural invasion is significantly correlated with both distant metastasis and unfavourable disease outcome [19]. Which requires more aggressive treatment for some authors in such cases [4]. The predominantly submucosal nature of these tumors makes early detection challenging, particularly in the subglottic region, where tumors can invade deeply before being diagnosed [6,18]. As a result, patients with laryngeal minor salivary gland tumors frequently present with advanced-stage disease (stage III or IV) [18].

Accurate preoperative imaging is essential for assessing both locoregional and distant tumor extension. On CT, ACC of the minor salivary glands typically appears as an extensive soft tissue mass with prominent submucosal spread [18]. CT is particularly useful in detecting involvement of adjacent cartilage, such as erosion or destruction, especially in cases of tracheal extension and cricoid cartilage invasion in subglottic tumors [10,18].

MRI imaging features are more discriminating than those of CT, particularly for accurately assessing tumor extension [18]. Additionally, analysis of signal characteristics can help to predicting the tumor’s histological subtype. T2WI hypointense lesions are typically associated with the solid type which is linked to a poorer prognosis, whereas hyperintense lesions are indicative of cribriform or tubular subtypes [10].

There is no universally accepted consensus on the optimal treatment strategy for laryngeal adenoid cystic carcinoma (ACC). However, due to the tumor's propensity for submucosal spread, perineural invasion, and lymphovascular involvement, surgery followed by radiotherapy is widely recommended as the standard treatment approach. This combined modality is considered superior to either surgery or radiotherapy alone [1,2,6,10,17]. Dubal et al. reported that patients who underwent surgical resection had a 73.7% survival rate, significantly higher than the 44.4% observed in those who did not receive surgery [14]. This finding highlights the importance of early and aggressive surgical intervention in the management of this lesion [14].

A total laryngectomy is frequently necessary and is widely regarded as the optimal treatment to achieve wide-margin resection [6]. However partial laryngectomy may be feasible in selected cases with small, well-defined tumors and negative surgical resection margins [6]. Lionello et al. recommend conservative surgery whenever possible, in order to preserve voice function, and reserve total laryngectomy for cases with massive extra laryngeal spread, or in the setting of local recurrence [3]. In addition, the rate of local recurrence and distant metastases do not appear to be significantly affected by the surgical approach used (conservative or radical) [13].

Therefore, the most conservative surgery possible should be chosen, tailored to the extent of the tumor and the patient's condition [13]. While total laryngectomy is often required for subglottic LACC, the surgical approach for supraglottic tumors depends largely on the extent of invasion into adjacent structures [1].

Lymph node metastasis is uncommon in adenoid cystic carcinoma (ACC), therefore the guidelines for neck dissection are unclear. Some studies have reported that patients who underwent surgery combined with elective neck dissection experienced longer periods without metastasis, or regional recurrence; however, no significant impact on overall survival or final outcome was observed [4,16].

Many authors recommend that neck dissection in laryngeal ACC (LACC) should be reserved for patients with clinical and/or radiological evidence of nodal involvement, or in cases of lymphovascular invasion, solid histopathologic subtype or rare cases of HGT-AdCC (Adenoid Cystic Carcinoma with Transformation to High Grade) [3,9,12,16].

Given the low incidence of occult neck metastases in ACC patients (approximately 17%) and the lack of survival benefit or difference in event-free survival (EFS) between patients who underwent elective neck dissection (END) and those who did not, systematic neck dissection should not be performed in clinically node-negative (cN0) patients [7,15].

Postoperative radiotherapy may serve an adjuvant treatment to surgery, particularly in cases with perineuronal spread, positive surgical margins, high-grade tumors, or clinical/radiological evidence of nodal metastases [6,3]. It has been shown to be effective in improving local disease control and tumor-free survival [14,2,17]. However, Mur et al reported no significant difference in overall survival between patients treated with surgery alone and those treated with surgery followed by radiotherapy [11]. The role of chemotherapy in ACC remains uncertain; it may be indicated as palliation with radiotherapy or in combination with surgery to prevent distant metastases [1,6].

For our patient we opted for a total laryngectomy without neck dissection followed by adjuvant radiotherapy given the cN0 status and presence of perineural invasion. Unfortunately, the patient was lost to follow-up and did not complete the radiotherapy sessions.

Distant metastases are common in ACC and may occur several years after the initial diagnosis and treatment. They often represent the leading cause of mortality in these patients [6,9]. Therefore, regular, close, and long-term follow-up is essential for the early detection of recurrence and distant metastases [6]."

Based on this review, total laryngectomy appears to be the preferred treatment for laryngeal adenoid cystic carcinoma, whereas conservative surgery may be considered in selected cases with limited disease. Lymph node dissection should not be performed systematically in clinically node-negative (cN0) patients. Radiotherapy is reserved for cases with perineural invasion, positive margins and high-grade tumors. Ultimately, the optimal treatment strategy should be determined on a case-by-case basis by a multidisciplinary team.

## Conclusion

Adenoid cystic carcinoma of the larynx is a rare malignant tumor. most commonly arising in the subglottic or glottic region. Surgical resection remains the cornerstone of treatment either alone or followed by adjuvant therapy. The decision between partial and total laryngectomy as well as the precise role of radiotherapy, remains a subject of ongoing discussion. Regular and long-term follow-up is mandatory, in order to detect recurrence and metastasis.

## Patient consent

Written informed consent was obtained from the patient for publication of this case report and accompanying images. A copy of the written consent is available for review by the Editor-in-Chief of this journal on request.

## CRediT authorship contribution statement

Fatima Zahra Essahli: Is the first author, study concept or design, data collection, data analysis or interpretation, writing the paper. Achraf Amine Sbai: study concept or design, data collection, data analysis or interpretation, writing the paper. Anass Haloui: Interpretation of histological data. Amal Benani: Confirm the histological diagnosis. Drissia Benfadil: participated in the writing, supervised, and revised the manuscript. Adil abdenbi Tsen: participated in the writing, supervised, and revised the manuscript, Azzedine Lachkar: supervised and revised the final manuscript. Fahd Elayoubi: supervised and revised the final manuscript.
